# NeuroGaze: a hybrid EEG and eye-tracking brain-computer interface for hands-free interaction in virtual reality

**DOI:** 10.3389/fnhum.2025.1695446

**Published:** 2025-11-21

**Authors:** Kyle Coutray, Wanyea Barbel, Zack Groth, Joseph J. LaViola

**Affiliations:** Department of Computer Science, Interactive Systems and User Experience Research Cluster, University of Central Florida, Orlando, FL, United States

**Keywords:** brain-computer interface (BCI), electroencephalography (EEG), eye tracking, virtual reality (VR), hybrid interfaces, hands-free interaction, human-computer interaction (HCI), accessibility

## Abstract

Brain-Computer Interfaces (BCIs) have traditionally been studied in clinical and laboratory contexts, but the rise of consumer-grade devices now allows exploration of their use in daily activities. Virtual reality (VR) provides a particularly relevant domain, where existing input methods often force trade-offs between speed, accuracy, and physical effort. This study introduces NeuroGaze, a hybrid interface combining electroencephalography (EEG) with eye tracking to enable hands-free interaction in immersive VR. Twenty participants completed a 360° cube-selection task using three different input methods: VR controllers, gaze combined with a pinch gesture, and NeuroGaze. Performance was measured by task completion time and error rate, while workload was evaluated using the NASA Task Load Index (NASA-TLX). NeuroGaze successfully supported target selection with off-the-shelf hardware, producing fewer errors than the alternative methods but requiring longer completion times, reflecting a classic speed-accuracy tradeoff. Workload analysis indicated reduced physical demand for NeuroGaze compared to controllers, though overall ratings and user preferences were mixed. While the differing confirmation pipelines limit direct comparison of throughput metrics, NeuroGaze is positioned as a feasibility study illustrating trade-offs between speed, accuracy, and accessibility. It highlights the potential of consumer-grade BCIs for long-duration use and emphasizes the need for improved EEG signal processing and adaptive multimodal integration to enhance future performance.

## 1 Introduction

Virtual reality (VR) systems have advanced rapidly in terms of visual immersion and motion tracking, but interaction remains a central challenge (Jerald, 2015; LaViola Jr. et al., 2017; Chong et al., 2018). The input devices that mediate user actions fundamentally shape the quality of the experience, and each current method carries trade-offs. Handheld controllers remain the most common solution, offering speed and precision through ray casting and button presses. However, extended use of controllers can be fatiguing, particularly in tasks that require repetitive pointing or in scenarios where hands-free interaction is desirable (Meier et al., 2021). Gaze-based dwell selection has been proposed as a more natural, ergonomic alternative (Sidenmark et al., 2022) where users fixate on a target and selection occurs after a brief dwell time, building on decades of research into the fundamental dynamics of eye movements (Martinez-Conde and Macknik, 2008; Cannon, 1992). While hands-free, this approach is slower, vulnerable to the “Midas touch” problem (Tang et al., 2025) of unintended activations, and can feel unnatural when prolonged fixations are required (Mohan et al., 2018; Isomoto et al., 2018; Chakraborty et al., 2014). More recently, combinations of eye tracking with manual gestures, such as pinch confirmation, have improved speed and reduced false selections (Zhang et al., 2020; Vertegaal, 2008; Stellmach and Dachselt, 2012). Yet these methods still depend on reliable hand mobility and introduce motor demands that limit accessibility for some users (Gherman et al., 2018).

Brain-Computer Interfaces (BCIs) offer an intriguing pathway to augment VR interaction by providing a neural channel for intent confirmation (Saxena et al., 2024). Traditionally restricted to clinical and tightly controlled experimental contexts (Padfield et al., 2019; Nicolas-Alonso and Gomez-Gil, 2012), BCIs are now becoming accessible outside the lab with the rise of consumer-grade headsets and biosensors. These devices make it feasible to test interaction techniques not only in laboratory studies but also in the context of daily activities (Vasiljevic and de Miranda, 2020; Pan et al., 2017; Rizzo et al., 2024). Prior research has demonstrated the feasibility of integrating electroencephalography (EEG) with eye tracking for selection tasks in desktop environments and experimental prototypes (Putze et al., 2013, 2016; Hild et al., 2014). For example, hybrid EEG+gaze systems have been used to disambiguate visual targets, detect covert attention, or reduce false activations (Shishkin et al., 2016; Kalaganis et al., 2018; Vortmann et al., 2022; Évain et al., 2016). However, most of this work has remained confined to controlled laboratory setups or 2D displays, with limited exploration in fully immersive VR environments (Larsen et al., 2024) and little emphasis on consumer-grade hardware. As a result, the real-world practicality of such systems for daily activities remains uncertain. The present work therefore serves as a proof-of-concept feasibility assessment rather than a full empirical evaluation.

To address this gap, this study introduces and assesses the feasibility of NeuroGaze, a hybrid EEG and eye-tracking interface designed for immersive VR using readily available consumer devices (Meta Quest Pro for eye tracking and Emotiv EPOC X for EEG). Unlike prior work that has focused narrowly on proof-of-concept demonstrations, this study conducts a focused feasibility comparison of NeuroGaze against two widely adopted VR input methods: hand controllers and eye tracking with a pinch hand gesture. In doing so, we provide one of the first comparative validations of a consumer-grade hybrid EEG+gaze system in immersive VR. While the tested input modalities necessarily differ in their confirmation mechanisms (e.g., button press vs. EEG classifier response), this evaluation emphasizes feasibility and maps the trade-offs between completion time, accuracy, and physical effort. In this way, NeuroGaze is positioned within the broader design space of VR interaction, particularly for contexts that prioritize accessibility and low physical demand.

## 2 Materials and methods

### 2.1 Participants

Twenty healthy adult volunteers (12 male, 8 female; age range 18–32 years) were recruited from the university community. All participants reported normal or corrected-to-normal vision, no history of neurological or motor impairments, and no susceptibility to simulator sickness. Inclusion criteria required participants to be at least 18 years of age, proficient in English, and physically able to wear both the EEG headset and the VR head-mounted display.

Participants represented a broad range of prior VR experience, from no exposure to frequent recreational use. Approximately 25% of the sample reported little or no prior VR experience, 60% reported moderate to above-moderate experience, and 15% described themselves as very experienced. Comparable distributions were observed for AR exposure and VR gaming, indicating that the sample encompassed both novices and highly experienced users.

All participants provided written informed consent prior to participation. The study was approved by the university's Institutional Review Board (IRB ID: STUDY00006401).

### 2.2 Apparatus

**Figure 1:**
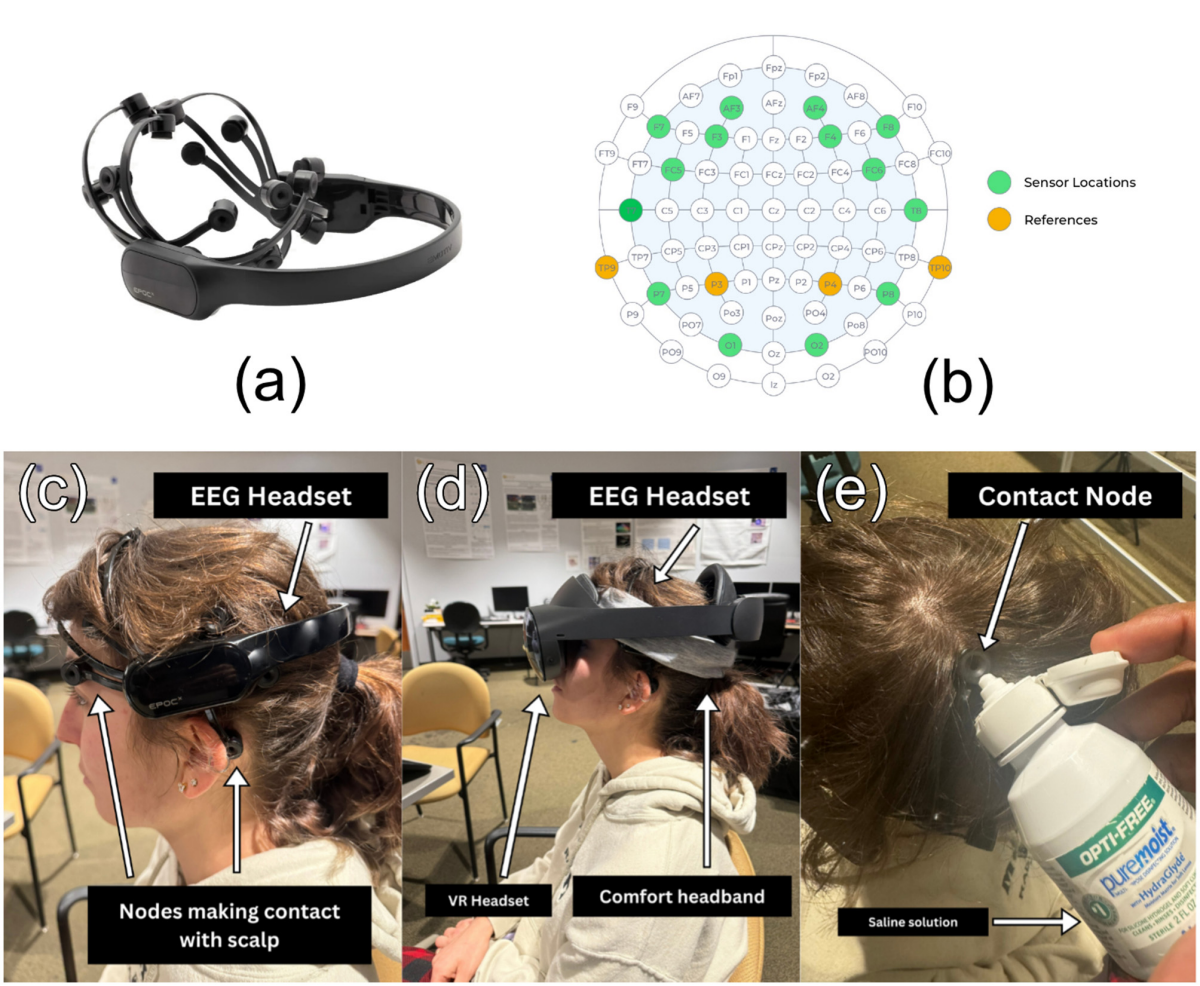
**(a)** The Emotiv EPOC X headset used for EEG data collection. **(b)** Electrode montage showing the 14 active sensor locations (green) and mastoid reference electrodes (orange) based on the international 10–20 system. **(c)** Emotiv EPOC X EEG headset with electrodes in contact with the scalp. **(d)** Combined configuration of the EPOC X, comfort headband, and Meta Quest Pro VR headset worn simultaneously. **(e)** Application of saline solution to EPOC X electrodes to maintain stable contact quality.

The immersive environment was presented using a Meta Quest Pro head-mounted display (Meta Platforms Inc., USA) with integrated binocular eye tracking. The headset provided real-time gaze vectors at a sampling rate of 72 Hz (Hou et al., 2024), and participants completed a standard five-point calibration at the beginning of each session. EEG activity was recorded using an Emotiv EPOC X headset (Emotiv Inc., USA), which features 14 active electrodes positioned according to the international 10–20 system (Khazi et al., 2012) (AF3, F7, F3, FC5, T7, P7, O1, O2, P8, T8, FC6, F4, F8, and AF4) with mastoid references (TP9, P3, P4, TP10) shown in Figures 1a, b. EEG signals were captured internally at 2,048 Hz, then downsampled to 128 Hz for wireless transmission via Bluetooth Low Energy (Emotiv Inc., 2020). Electrode-skin contact quality was continuously monitored, with saline solution (OPTI-FREE) reapplied as needed to maintain stable impedance. The NeuroGaze setup required participants to wear both the Emotiv EPOC X and Meta Quest Pro simultaneously, often secured with a comfort headband to ensure reliable electrode contact (Figures 1c–e).

**Figure 2:**
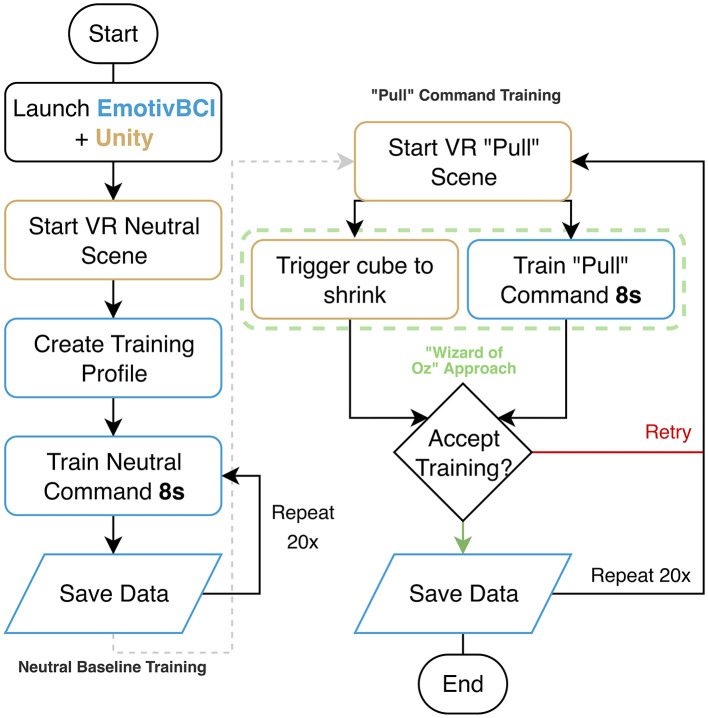
Flowchart of the NeuroGaze EEG calibration procedure. The process consists of two stages: neutral mental command training **(left)**, where participants repeatedly train a relaxed state in the EmotivBCI program, and “pull” mental command training **(right)**, where participants attempt to imagine a “pull” action while the Unity engine triggers object shrinkage through a “Wizard-of-Oz” approach. Each command was trained in 20 repetitions, with accepted trials saved to the user's training profile for later classification during the experiment.

The experimental software was developed in Unity (Unity Technologies, USA) using the Meta XR All-in-One SDK. Eye tracking was used to control a visual ray pointer and object hover state, rendered as a white line from the midpoint of the user's eyes to 500 meters in the forward direction. This ray cast triggered a scaling effect on the interactable cube objects, causing them to grow to a fixed scale (0.2304m^3^) when hovered over and shrink back (0.18m^3^) when not. EEG signals were streamed into Unity through the Emotiv Cortex API. EEG calibration involved training two mental command classes: a neutral state (representing relaxed, unfocused brain activity) and a “pull” command associated with selection. During calibration, participants viewed objects that appeared and shrank in synchrony with their imagined action, providing feedback to reinforce consistent neural patterns (this was achieved through a Wizard-of-Oz approach in which the experiment administrator manually triggered the object to shrink as seen in Figure 2). Once trained, the classifier output was integrated into the Unity selection loop: objects under gaze became eligible for interaction, and a detected pull command triggered selection. The EmotivBCI program handled training profiles, EEG noise sanitization, and classification of EEG artifacts. The EEG classifier stream was linked directly to Unity's gaze-ray events, enabling synchronized neural and visual input handling within a unified control loop. To maintain temporal alignment between modalities, Unity event markers were logged concurrently with EEG data via the Emotiv Cortex API on the same host system.

### 2.3 Task

**Figure 3:**
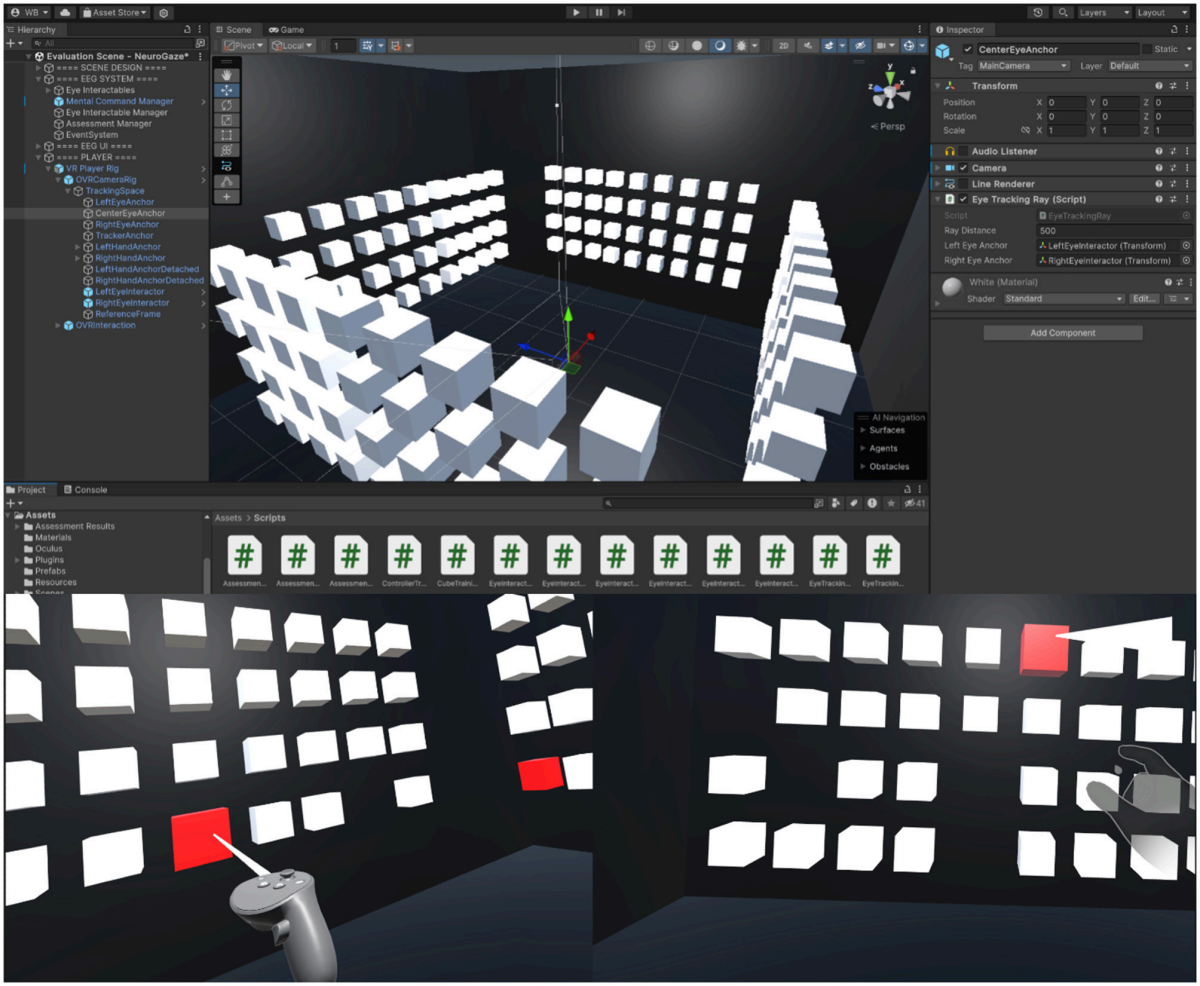
**Top**: Unity editor view of the 360° object-selection task environment, with four cube arrays surrounding the participant. **Bottom**: Example participant perspectives during task execution. **Left**: VR Controller (VRC) condition; **Right**: Eye Gaze + Hand Gesture (EG+HG) condition.

Participants completed a 360° object-selection task in a virtual environment (VE). The environment consisted of four surrounding walls, each displaying a 4 × 9 array of white cubes (36 per wall; 144 total) as seen in Figure 3.

At the start of each block, 12 random cubes (three per wall) were designated as targets by turning red. Participants were instructed to select these targets as quickly and accurately as possible. When a target was successfully selected, it disappeared from the scene, and the block concluded once all targets had been cleared. Each input condition (VRC, EG+HG, and NG) was performed across three blocks, for a total of nine task blocks per participant. The average distance from the user to each wall of cubes was approximately 2 m.

The task required participants to rotate their heads and bodies to engage with stationary targets distributed across the 360° field.

### 2.4 Experimental conditions

Each participant completed the selection task under three input modalities. The order of conditions was randomized across participants to minimize order and learning effects.

**VR controllers (VRC):** Participants used standard handheld VR controllers to interact with the virtual environment. A ray projected from the end of the controller was used to aim at targets, with selection confirmed via a trigger button press. This condition represented the conventional VR input method and served as the baseline for speed and precision.

**Eye Gaze** **+** **Hand Gesture (EG+HG):** Participants fixated on target cubes using the Quest Pro's integrated eye-tracking, confirming selections with a pinch gesture detected by the headset's optical hand tracking. This provided gaze-driven aiming with explicit manual confirmation, similar to interaction paradigms in emerging AR headsets.

**NeuroGaze (NG):** Participants aimed using eye gaze, with selection confirmed by an EEG-based “pull” mental command classified in real time by the EmotivBCI software from signals acquired by the Emotiv EPOC X headset. This condition provided a fully hands-free interaction method that relied on EEG signal processing for confirmation, inherently involving greater computational latency than the other input modes.

### 2.5 Measures

Task performance was evaluated using two behavioral measures. Completion time was defined as the total duration (in seconds) from target onset to the confirmed selection of all 12 red cubes, as recorded in Unity environment logs. Error rate was defined as the proportion of incorrect or missed selections relative to the total number of targets per block, encompassing both missed and unintended object selections.

Subjective workload was evaluated after each condition using the NASA Task Load Index (NASA-TLX), which provides ratings across six sub-scales: Mental Demand, Physical Demand, Temporal Demand, Performance, Effort, and Frustration. To derive the overall workload score, the sub-scales were combined according to Equation 1.


$$\begin{array}{l} \text{NASA-TLX}=\text{Mental Demand}+\text{Physical Demand}\\ \;+\text{Temporal Demand}+\left(7-\text{Performance}\right)\\ \;+\text{Effort}+\text{Frustration} \end{array}$$ (1)

Both aggregated NASA-TLX scores and individual sub-scale ratings were retained for analysis.

Although EEG and eye-tracking data were continuously streamed, the present analysis focused on behavioral performance and subjective workload (NASA-TLX) in keeping with the feasibility scope of this study. Objective physiological indices of cognitive or visual load were not computed due to processing complexity and the low signal-to-noise ratio of consumer-grade hardware.

Finally, overall preference was captured through a post-experiment ranking task. After completing all three input conditions, participants ranked the modalities from most preferred (rank = 1) to least preferred (rank = 3). This ranking provided a simple comparative index of participants' subjective impressions of each input method.

### 2.6 Analysis plan

Task completion time was analyzed with a repeated-measures design. Mauchly's test of sphericity was first applied; if violations were detected (*p* < 0.001), Greenhouse-Geisser corrections were used. A repeated-measures ANOVA was then conducted with Input Condition (VRC, EG+HG, NeuroGaze) as the within-subjects factor. Significant effects were followed up with Bonferroni-corrected pairwise t-tests. Effect sizes (partial η^2^) were reported alongside significance values. Pairwise contrasts were followed by computation of 95% confidence intervals and corresponding effect sizes (*d* or *r*), reported for each comparison to provide standardized measures of difference magnitude and directionality.

Error rates were analyzed similarly. Mauchly's test indicated that the assumption of sphericity was met (*p* = 0.85), so a repeated-measures ANOVA was conducted on average error counts. *Post-hoc* comparisons were performed with paired t-tests, and η^2^ effect sizes were reported.

Subjective workload was evaluated using NASA-TLX ratings. Aggregated workload scores were compared across conditions using a Friedman test. Individual sub-scales (Mental Demand, Physical Demand, Temporal Demand, Performance, Effort, and Frustration) were analyzed with Wilcoxon signed-rank tests. Bonferroni correction was applied, setting the adjusted threshold for significance at α = 0.0157.

User preference rankings were analyzed with a Chi-squared test of independence to examine associations between input modality and rank position. Visualizations were prepared to illustrate group-level performance, including bar plots for completion time, error rate, and NASA-TLX scores.

## 3 Results

The results are organized into three subsections corresponding to the main dependent measures: task completion time, error rate, and subjective workload. Statistical analyses were performed using repeated-measures designs with Condition (VRC, EG+HG, NeuroGaze) as the within-subjects factor. All reported pairwise comparisons used Bonferroni-corrected p-values, and effect sizes are presented alongside significance values.

### 3.1 Completion time

Task completion time differed significantly across input conditions. Mauchly's test indicated that sphericity was not violated, χ^2^(2) = 5.766, *p* = 0.056, ε_*GG*_ = 0.785. A repeated-measures ANOVA revealed a robust main effect of condition, *F*(2, 38) = 275.4, *p* < 0.001, $\eta_{p}^{2}\;=\;0.935$.

**Figure 4:**
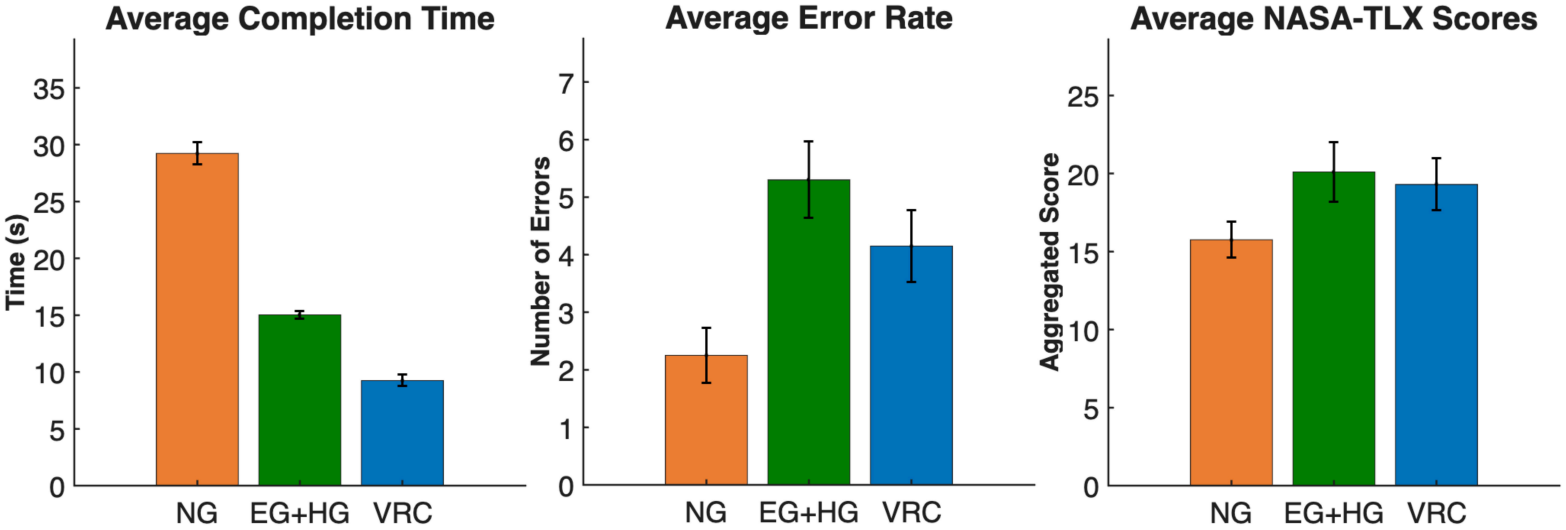
Task performance and subjective workload across input devices. **Left**: Average task completion time (s). **Middle**: Average error count. **Right**: Aggregated NASA-TLX workload scores. Conditions are labeled as follows: EG+HG, Eye Gaze + Hand Gesture; VRC, VR Controllers; NG, NeuroGaze. Error bars represent 95% confidence intervals.

Participants completed the selection task fastest with VR Controllers (*M* = 9.25 s, *SD* = 2.26 s), followed by Eye Gaze + Hand Gesture (*M* = 15.02 s, *SD* = 1.61 s), and slowest with NeuroGaze (*M* = 29.23 s, *SD* = 4.33 s) (Figure 4). Pairwise comparisons confirmed that both VR Controllers [*t*_(19)_ = 20.98, *p* < 0.001, *d* = 4.69, 95% CI (3.14, 6.23)] and Eye Gaze + Hand Gesture [*t*_(19)_ = 14.03, *p* < 0.001, *d* = 3.14, 95% CI (2.05, 4.21)] were significantly faster than NeuroGaze. VR Controllers were also significantly faster than Eye Gaze + Hand Gesture [*t*_(19)_ = 9.47, *p* < 0.001, *d* = 2.12, 95% CI (1.31, 2.91)].

This pattern indicates that while NeuroGaze enabled reliable hands-free selection, its current implementation introduced substantial latency compared to standard input methods.

### 3.2 Error rate

Error rates differed significantly across input conditions. A repeated-measures ANOVA (Mauchly's test indicated sphericity was met: χ^2^(2) = 1.291, *p* = 0.524) revealed a main effect of condition, *F*_(2, 38)_ = 6.27, *p* = 0.004, $\eta_{p}^{2}\;=\;0.25$.

On average, participants made the fewest errors with NeuroGaze (*M* = 2.25, *SD* = 2.12), followed by VR Controllers (*M* = 4.15, *SD* = 2.78) and Eye Gaze + Hand Gesture (*M* = 5.30, *SD* = 2.98) (Figure 4). Pairwise comparisons showed that NeuroGaze resulted in significantly fewer errors than Eye Gaze + Hand Gesture [*t*_(19)_ = 3.18, *p* = 0.005, *d* = 0.71, 95% CI (0.21, 1.20)]. Differences between NeuroGaze and VR Controllers [*t*_(19)_ = 1.52, *p* = 0.146, *d* = 0.34, 95% CI (–0.12, 0.79)] and between VR Controllers and Eye Gaze + Hand Gesture [*t*_(19)_ = −2.15, *p* = 0.044, *d* = −0.48, 95% CI (–0.94, –0.01)] were not significant after correction.

These results indicate that participants made significantly fewer errors when using NeuroGaze compared to Eye Gaze + Hand Gesture, while differences between other pairs did not reach significance.

### 3.3 NASA-TLX

Subjective workload ratings from the NASA-TLX revealed differences across conditions, although patterns varied by sub-scale. Aggregated workload scores did not differ significantly between modalities (VRC: M = 19.30; EG+HG: M = 20.10; NeuroGaze: M = 15.75), Friedman χ^2^(2) = 0.29, *p*>0.05 (Figure 4).

When sub-scales were examined individually using Wilcoxon signed-rank tests with Bonferroni correction (α = 0.0157), several distinctions emerged. Physical Demand was lowest for NeuroGaze (*M* = 2.4), which was significantly lower than Eye Gaze + Hand Gesture (*M* = 3.8, *p* = 0.006). NeuroGaze also trended lower than VR Controllers (*M* = 3.85, *p* = 0.020), but this did not survive correction. No difference was observed between Eye Gaze + Hand Gesture and VR Controllers (*p* = 0.943).

Temporal Demand showed the strongest effects. NeuroGaze (*M* = 2.4) was rated significantly less demanding than both Eye Gaze + Hand Gesture (*M* = 3.6, *p* = 0.002) and VR Controllers (*M* = 4.35, *p* = 0.001). Eye Gaze + Hand Gesture trended higher than VR Controllers (*p* = 0.028) but did not reach the adjusted threshold.

For Mental Demand, no significant differences were found. NeuroGaze (*M* = 3.2) did not differ from either Eye Gaze + Hand Gesture (*p* = 0.129) or VR Controllers (*p* = 0.840); however, Eye Gaze + Hand Gesture appeared slightly more demanding than VR Controllers (*p* = 0.028), representing a weak trend.

No significant differences emerged for Perceived Performance, Effort, or Frustration. Several comparisons trended toward significance but did not survive Bonferroni correction (α = 0.0157): for Perceived Performance, Eye Gaze + Hand Gesture (*M* = 4.75) tended to be rated lower than VR Controllers (*p* = 0.040). For Effort and Frustration, no comparisons approached significance (*p* > 0.15).

Overall, NeuroGaze was associated with lower physical and temporal demand relative to the other modalities, confirming its ergonomic advantage but showing no reliable differences in cognitive or affective workload dimensions.

### 3.4 User preference

After completing all three conditions, participants ranked the input modalities by overall preference. NeuroGaze was most often ranked first (10 participants), followed by VR Controllers (5) and Eye Gaze + Hand Gesture (5). Intermediate rankings were more evenly distributed (VR Controllers: 8; NeuroGaze: 6; Eye Gaze + Hand Gesture: 6). Least-preferred rankings were most frequently assigned to Eye Gaze + Hand Gesture (9 participants), followed by VR Controllers (7) and NeuroGaze (4).

A chi-squared test of independence revealed no significant association between input modality and preference ranking, χ^2^(2, *N* = 20) = 4.8, *p* = 0.31.

Qualitative feedback provided additional context. VR Controllers were praised for their speed and familiarity. Eye Gaze + Hand Gesture was described as intuitive but often unreliable, with several participants noting difficulty executing pinch gestures. NeuroGaze was appreciated for its novelty and hands-free interaction, though some participants reported discomfort from wearing both headsets and noted slower response times.

## 4 Discussion

### 4.1 Interpretation

The findings highlight both the strengths and limitations of current VR input modalities. Handheld controllers remain the benchmark for speed, with participants consistently achieving the fastest completion times. This reflects both the maturity of the technology and its optimization for rapid, precise selection tasks. Eye tracking with pinch gestures (EG+HG) occupied an intermediate position, offering more intuitive aiming than controllers but prone to gesture-recognition errors.

In contrast, NeuroGaze yielded fewer errors overall, but this advantage came at the cost of substantially slower task completion times. Accuracy appears to result less from superior input fidelity than from participants' conservative pacing, further amplified by the processing delay inherent in EEG classification, representing a clear example of the classic speed-accuracy tradeoff. The longer completion times observed for NeuroGaze (M = 29.23 s) relative to VR Controllers (M = 9.25 s) stem primarily from the processing and decision latency intrinsic to consumer-grade EEG hardware (Emotiv EPOC X), which necessitated a binary command scheme and conservative activation thresholds to ensure reliability. Consequently, these timing differences reflect the design constraints and confirmation mechanisms of each modality rather than a direct measure of throughput competitiveness. Thus, while NG does not yet rival existing VR inputs on raw performance metrics, it provides a viable, hands-free alternative that emphasizes ergonomic accessibility (Hhne et al., 2014; Kos'myna and Tarpin-Bernard, 2013) rather than speed. Rather than competing with controllers in high-speed applications, NG is better suited to daily-activity contexts where comfort, inclusivity, and error minimization are paramount.

Workload ratings reinforce this role. NeuroGaze reduced physical and temporal demand relative to other inputs while maintaining similar cognitive workload. Despite slower operation, its lower effort and fatigue suggest advantages for accessibility and extended-use contexts.

### 4.2 Contribution

This study provides one of the first systematic evaluations of a consumer-grade hybrid EEG and eye-tracking system benchmarked against established VR input modalities in a fully immersive environment. Prior work on gaze-EEG interaction has largely relied on laboratory hardware or 2D displays, limiting ecological validity and applicability to everyday contexts. By deploying NeuroGaze with widely available devices—the Meta Quest Pro and Emotiv EPOC X—this study shows that hybrid brain-computer interfaces are no longer confined to specialized laboratories and can be assessed under conditions closer to daily VR use.

Benchmarking NeuroGaze against two established input modalities (controllers and gaze+pinch) further clarified its comparative strengths and weaknesses. While slower than conventional inputs, NeuroGaze offers a tangible ergonomic benefit, demonstrated by lower physical demand ratings and fully hands-free operation. These qualities suggest that the system is not a competitor to controllers in time-sensitive or performance-critical contexts, but rather a complementary modality where accessibility, comfort, and reduced fatigue are prioritized.

The most promising applications of NeuroGaze may therefore lie in daily-activity and accessibility-oriented scenarios that demand sustained interaction without physical strain (Sellers et al., 2010). Examples include VR-based rehabilitation, training for individuals with motor impairments, or prolonged use cases where repetitive arm or hand motions become burdensome. By reframing the role of BCIs away from speed competition and toward ergonomic inclusivity, this study contributes to a broader vision of BCIs as practical tools for everyday human-computer interaction.

### 4.3 Limitations

Several limitations of the present study should be acknowledged. The sample size was modest (*N* = 20), limiting generalizability and reducing statistical power to detect subtle effects. While adequate for a brief feasibility study, larger samples are needed to obtain more stable estimates of performance across diverse populations.

The task design employed static targets arranged across four walls, providing consistency but not capturing the dynamic and unpredictable environments typical of VR. Future work should incorporate moving or context-sensitive stimuli to assess real-world applicability.

The EEG calibration procedure incorporated a Wizard-of-Oz component in which feedback was artificially reinforced to improve classifier training. Although the actual task relied on trained classifiers, this approach may have inflated participants' perception of system reliability during calibration.

Use of consumer-grade EEG hardware (Emotiv EPOC X) imposed signal-to-noise and artifact limitations, restricting the system to a binary command scheme and limiting classifier sophistication.

A methodological consideration is that the compared input modalities had intrinsically different confirmation latencies: VR Controllers provided instantaneous input, Eye Gaze + Hand Gesture introduced optical tracking delay, and NeuroGaze added EEG processing time. Thus, completion times reflect cumulative latency rather than normalized throughput. Without detailed metrics such as Information Transfer Rate (ITR) or speed-accuracy operating curves, the behavioral comparison should be interpreted as a feasibility assessment rather than a performance benchmark. Future studies should include throughput-oriented paradigms such as Fitts' Law tasks.

Participants were primarily young, tech-literate individuals (approximately 75% with prior VR experience), predisposing them toward conventional input methods. This familiarity may have influenced both performance and preference ratings. The greatest value of hands-free interaction likely lies in populations with reduced motor control or fatigue sensitivity, which were not represented here. Future evaluations should include users with motor impairments to assess NeuroGaze's translational potential for inclusive, low-effort interaction.

Finally, ergonomic incompatibility between the EEG headset and the Meta Quest Pro caused minor discomfort during extended use. Although commercial devices enhance ecological validity, they also constrain neural input fidelity and overall user comfort.

### 4.4 Future work

Several avenues for future development emerge from the present findings. Future iterations of NeuroGaze should advance toward more responsive, fully contactless brain control through tighter integration of hardware and software components. A key priority is the reduction of system latency. NeuroGaze's slower performance relative to traditional input methods reflects both the computational overhead of EEG signal classification and the conservative thresholds used to minimize false activations. Advances in machine learning and signal processing—such as adaptive filtering, transfer learning across users, and real-time artifact rejection—may help reduce response times while maintaining accuracy, thereby improving the practical viability of hybrid BCI input. Another promising direction involves adaptive multimodal switching, in which NeuroGaze could dynamically integrate with conventional controllers or gesture-based systems. For example, users might rely on EEG+gaze input for sustained, low-effort interaction but seamlessly transition to controller-based input when speed or fine-grained control is required. Such hybrid workflows would leverage the strengths of each modality and broaden the contexts in which BCIs are practical. Future systems might also incorporate complementary biosignals (e.g., fNIRS, EMG, pupillometry) to improve robustness and expand command vocabulary. Finally, future studies should move beyond healthy young adults to evaluate NeuroGaze in accessibility scenarios. Populations with motor impairments, fatigue-related conditions, or limited hand mobility stand to benefit most from hands-free BCI interaction. Assessing usability, comfort, and performance in these groups will be essential for determining NeuroGaze's translational potential in rehabilitation, assistive technology, and daily activity contexts.

## 5 Conclusion

This study introduced and evaluated NeuroGaze, a hybrid EEG and eye-tracking interface implemented with consumer-grade hardware in an immersive VR environment. Compared to conventional controllers and gaze+pinch interaction, NeuroGaze enabled reliable, fully hands-free object selection, though at the cost of slower task completion times. The results reflect a classic speed-accuracy tradeoff: participants made fewer errors with NeuroGaze, but this advantage stemmed largely from more deliberate pacing rather than inherently superior input fidelity. Despite these performance constraints, NeuroGaze demonstrates clear ergonomic and accessibility promise. By reducing physical demand and eliminating the need for handheld controllers, it extends VR interaction beyond speed-driven contexts toward scenarios where comfort, inclusivity, and reduced fatigue are prioritized. Rather than serving as a replacement for controllers in time-critical tasks, NeuroGaze should be considered a complementary modality for daily activities, rehabilitation contexts, and fatigue-sensitive environments where minimizing physical effort is critical. Taken together, these findings establish NeuroGaze as a feasibility demonstration of hybrid EEG+gaze interaction in immersive VR using readily available consumer devices. Future iterations should aim to reduce confirmation latency from the current multi-second range toward sub-second response times through improved signal processing, classifier optimization, and multimodal fusion. More broadly, the results highlight the potential of consumer-grade BCIs not as competitors to established input methods but as enablers of more inclusive and adaptable human-computer interaction.

## Data Availability

The datasets presented in this study can be found in online repositories. The names of the repository/repositories and accession number(s) can be found below: https://github.com/Wanyea/NeuroGaze/.

## References

[B1] Barbel W. (2024). Neurogaze in virtual reality: Assessing an EEG and eye tracking interface against traditional virtual reality input devices. Master's thesis, University of Central Florida, Orlando, FL. Graduate Thesis and Dissertation 2023–2024, STARS Digital Repository.

[B2] Cannon S. (1992). The neurology of eye movements (contemporary neurology series). Arch. Ophthalmol. 110, 326–326. doi: 10.1001/archopht.1992.01080150024016

[B3] Chakraborty T. Sarcar S. Samanta D. (2014). “Design and evaluation of a dwell-free eye typing technique,” in CHI '14 Extended Abstracts on Human Factors in Computing Systems, CHI EA '14 (New York, NY, USA: Association for Computing Machinery), 1573–1578. doi: 10.1145/2559206.2581265

[B4] Chong H. T. Lim C. K. Tan K. L. (2018). Challenges in virtual reality system: a review. AIP Conf. Proc. 2016:020037. doi: 10.1063/1.5055439

[B5] Emotiv Inc (2020). Emotiv EPOC X Technical Specifications. Internal sampling: 2048 Hz; downsampled to 128/256 Hz for BLE or other transmission.

[B6] Évain A. Argelaguet F. Casiez G. Roussel N. Lécuyer A. (2016). Design and evaluation of fusion approach for combining brain and gaze inputs for target selection. Front. Neurosci. 10:454. doi: 10.3389/fnins.2016.00454 27774048 PMC5054006

[B7] Gherman O. Schipor O. Gheran B.-F. (2018). “Verge: a system for collecting voice, eye gaze, gesture, and EEG data for experimental studies,” in 2018 International Conference on Development and Application Systems (DAS), 150–155. doi: 10.1109/DAAS.2018.8396088

[B8] Hhne J. Holz E. Staiger-Sälzer P. Müller K.-R. Kübler A. Tangermann M. (2014). Motor imagery for severely motor-impaired patients: evidence for brain-computer interfacing as superior control solution. PLoS ONE 9, 1–11. doi: 10.1371/journal.pone.0104854 25162231 PMC4146550

[B9] Hild J. Putze F. Kaufman D. Kühnle C. Schultz T. Beyerer J. (2014). “Spatio-temporal event selection in basic surveillance tasks using eye tracking and EEG,” in Proceedings of the 7th Workshop on Eye Gaze in Intelligent Human Machine Interaction: Eye-Gaze &Multimodality, GazeIn '14 (New York, NY, USA: Association for Computing Machinery), 3–8. doi: 10.1145/2666642.2666645

[B10] Hou B. J. Abdrabou Y. Weidner F. Gellersen H. (2024). “Unveiling variations: a comparative study of vr headsets regarding eye tracking volume, gaze accuracy, and precision,” in 2024 IEEE Conference on Virtual Reality and 3D User Interfaces Abstracts and Workshops (VRW), 650–655. doi: 10.1109/VRW62533.2024.00127

[B11] Isomoto T. Ando T. Shizuki B. Takahashi S. (2018). “Dwell time reduction technique using fitts' law for gaze-based target acquisition,” in Proceedings of the 2018 ACM Symposium on Eye Tracking Research &Applications, ETRA '18 (New York, NY, USA: Association for Computing Machinery). doi: 10.1145/3204493.3204532

[B12] Jerald J. (2015). The VR Book: Human-Centered Design for Virtual Reality. New York: Association for Computing Machinery and Morgan &Claypool.

[B13] Kalaganis F. P. Chatzilari E. Nikolopoulos S. Kompatsiaris I. (2018). An error-aware gaze-based keyboard by means of a hybrid BCI system. Sci. Rep. 8:13176. doi: 10.1038/s41598-018-31425-2 30181532 PMC6123473

[B14] Khazi M. Kumar A. Vidya M. (2012). Analysis of EEG using 10, 20 electrode system. Int. J. Innov. Res. Sci. Eng. Technol. 1, 185–191.

[B15] Kos'myna N. Tarpin-Bernard F. (2013). Evaluation and comparison of a multimodal combination of BCI paradigms and eye tracking with affordable consumer-grade hardware in a gaming context. IEEE Trans. Comput. Intell. AI Games 5, 150–154. doi: 10.1109/TCIAIG.2012.2230003

[B16] Larsen O. F. P. Tresselt W. G. Lorenz E. A. Holt T. Sandstrak G. Hansen T. I. . (2024). A method for synchronized use of EEG and eye tracking in fully immersive VR. Front. Hum. Neurosci. 18:1347974. doi: 10.3389/fnhum.2024.1347974 38468815 PMC10925625

[B17] LaViola J.r,. J. J Kruijff E. Bowman D. A. McMahan R. P. Poupyrev I. (2017). 3D User Interfaces: Theory and Practice. Boston: Addison-Wesley Professional.

[B18] Martinez-Conde S. Macknik S. L. (2008). Fixational eye movements across vertebrates: comparative dynamics, physiology, and perception. J. Vis. 8, 28–28. doi: 10.1167/8.14.28 19146329

[B19] Meier M. Streli P. Fender A. Holz C. (2021). “Demonstrating the use of rapid touch interaction in virtual reality for prolonged interaction in productivity scenarios,” in 2021 IEEE Conference on Virtual Reality and 3D User Interfaces Abstracts and Workshops (VRW), 761–762. doi: 10.1109/VRW52623.2021.00263

[B20] Mohan P. Goh W. B. Fu C.-W. Yeung S.-K. (2018). “Dualgaze: addressing the MIDAS touch problem in gaze mediated VR interaction,” in 2018 IEEE International Symposium on Mixed and Augmented Reality Adjunct (ISMAR-Adjunct), 79–84. doi: 10.1109/ISMAR-Adjunct.2018.00039

[B21] Nicolas-Alonso L. F. Gomez-Gil J. (2012). Brain computer interfaces, a review. Sensors 12, 1211–1279. doi: 10.3390/s120201211 22438708 PMC3304110

[B22] Padfield N. Zabalza J. Zhao H. Masero V. Ren J. (2019). EEG-based brain-computer interfaces using motor-imagery: techniques and challenges. Sensors 19:1423. doi: 10.3390/s19061423 30909489 PMC6471241

[B23] Pan P. Tan G. Wai A. A. P. (2017). “Evaluation of consumer-grade EEG headsets for bci drone control,” in Proceedings of the IRC Conference on Science, Engineering, and Technology.

[B24] Putze F. Hild J. Kärgel R. Herff C. Redmann A. Beyerer J. . (2013). “Locating user attention using eye tracking and EEG for spatio-temporal event selection,” in Proceedings of the 2013 International Conference on Intelligent User Interfaces, IUI '13 (New York, NY, USA: Association for Computing Machinery), 129–136. doi: 10.1145/2449396.2449415

[B25] Putze F. Popp J. Hild J. Beyerer J. Schultz T. (2016). “Intervention-free selection using EEG and eye tracking,” in Proceedings of the 18th ACM International Conference on Multimodal Interaction, ICMI '16 (New York, NY, USA: Association for Computing Machinery), 153–160. doi: 10.1145/2993148.2993199

[B26] Rizzo L. Zicari P. Cicirelli F. Guerrieri A. Micieli M. Vinci A. (2024). “A study on consumer-grade EEG headsets in BCI applications,” in 2024 IEEE Conference on Pervasive and Intelligent Computing (PICom), 67–74. doi: 10.1109/PICom64201.2024.00016

[B27] Saxena S. Ranjan M. K. Sattar A. M. (2024). “Brain-computer interfaces: a key to neural communication's limitless possibilities,” in 2024 1st International Conference on Cognitive, Green and Ubiquitous Computing (IC-CGU), 1–8. doi: 10.1109/IC-CGU58078.2024.10530664

[B28] Sellers E. W. Vaughan T. M. Wolpaw J. R. (2010). A brain-computer interface for long-term independent home use. Amyotr. Lateral Sclerosis 11, 449–455. doi: 10.3109/17482961003777470 20583947

[B29] Shishkin S. L. Nuzhdin Y. O. Svirin E. P. Trofimov A. G. Fedorova A. A. Kozyrskiy B. L. . (2016). EEG negativity in fixations used for gaze-based control: toward converting intentions into actions with an eye-brain-computer interface. Front. Neurosci. 10:528. doi: 10.3389/fnins.2016.00528 27917105 PMC5114310

[B30] Sidenmark L. Parent M. Wu C.-H. Chan J. Glueck M. Wigdor D. . (2022). Weighted pointer: Error-aware gaze-based interaction through fallback modalities. IEEE Trans. Vis. Comput. Graph. 28, 3585–3595. doi: 10.1109/TVCG.2022.3203096 36048981

[B31] Stellmach S. Dachselt R. (2012). “Look &touch: gaze-supported target acquisition,” in Proceedings of the SIGCHI Conference on Human Factors in Computing Systems, CHI '12 (New York, NY, USA: Association for Computing Machinery), 2981–2990. doi: 10.1145/2207676.2208709

[B32] Tang X. Chen X. Leng H. Wang Z. Chen B. Chen Y. . (2025). Comparison and optimization of target-assisted gaze input technique for enhanced selection in virtual eye-controlled systems. Int. J. Hum. Comput. Inter. 2025, 1–19. doi: 10.1080/10447318.2025.2505780

[B33] Vasiljevic G. A. M. de Miranda L. C. (2020). Brain–computer interface games based on consumer-grade EEG devices: a systematic literature review. Int. J. Hum. Comput. Inter. 36, 105–142. doi: 10.1080/10447318.2019.1612213

[B34] Vertegaal R. (2008). “A Fitts law comparison of eye tracking and manual input in the selection of visual targets,” in Proceedings of the 10th International Conference on Multimodal Interfaces, ICMI '08 (New York, NY, USA: Association for Computing Machinery), 241–248. doi: 10.1145/1452392.1452443

[B35] Vortmann L.-M. Ceh S. Putze F. (2022). Multimodal EEG and eye tracking feature fusion approaches for attention classification in hybrid BCIS. Front. Comput. Sci. 4:780580. doi: 10.3389/fcomp.2022.780580

[B36] Zhang S. Tian Y. Wang C. Wei K. (2020). Target selection by gaze pointing and manual confirmation: performance improved by locking the gaze cursor. Ergonomics 63, 884–895. doi: 10.1080/00140139.2020.1762934 32348191

